# Insulin and the sebaceous gland function

**DOI:** 10.3389/fphys.2023.1252972

**Published:** 2023-09-01

**Authors:** Obumneme Emeka Okoro, Emanuela Camera, Enrica Flori, Monica Ottaviani

**Affiliations:** ^1^ Dermatology Unit, Federal Medical Centre, Keffi, Nasarawa State, Nigeria; ^2^ Laboratory of Cutaneous Physiopathology and Integrated Center of Metabolomics Research, San Gallicano Dermatological Institute, IRCCS, Rome, Italy

**Keywords:** insulin, sebaceous gland, sebocytes, MTOR signaling, acne

## Abstract

Insulin affects metabolic processes in different organs, including the skin. The sebaceous gland (SG) is an important appendage in the skin, which responds to insulin-mediated signals, either directly or through the insulin growth factor 1 (IGF-1) axis. Insulin cues are differently translated into the activation of metabolic processes depending on several factors, including glucose levels, receptor sensitivity, and sebocyte differentiation. The effects of diet on both the physiological function and pathological conditions of the SG have been linked to pathways activated by insulin and IGF-1. Experimental evidence and theoretical speculations support the association of insulin resistance with acne vulgaris, which is a major disorder of the SG. In this review, we examined the effects of insulin on the SG function and their implications in the pathogenesis of acne.

## 1 Introduction

The cross-talk among different skin cell types is crucial in maintaining cutaneous homeostasis. These functional interactions are mediated by several, locally secreted factors including growth factors, small molecules, lipid mediators, and neuroendocrine agents (Picardo et al., 2015). In this context, the sebaceous gland (SG) activity has long been underestimated and SG has been considered “a living fossil of the skin” (Ligman, 1963). However, in recent times, the regulation of SG function has become one of the foci of scientific discussion in the field of dermatology for its relevant role in skin health and integrity (Melnik, 2015; Zouboulis et al., 2016; Zouboulis et al., 2020). The activities of the SG are controlled by various local and systemic stimuli mediated through a constellation of receptors expressed on the sebocytes (Krause et al., 2007; Pelle et al., 2008; Zouboulis, 2009). These factors not only affect the sebocyte function but also the pathomechanisms of various disorders arising from the gland. Acne vulgaris, a common disease affecting the pilo-sebaceous unit, is initiated and sustained by some of these stimuli (Dreno, 2017). Insulin, alongside the sibling hormone insulin-like growth factor-1 (IGF-1), is among the hormonal factors that affect the function of the SG (Deplewski and Rosenfield, 1999; Mirdamadi et al., 2015). They influence the metabolism of nutrients by the SG and are important players in the mechanisms of some of the diseases arising from the gland. In this review, we will touch upon the anatomy and physiology of the SG, the modes of action of insulin, and its effect on the SG function. Finally, the implication of insulin-mediated responses of the SG will be considered in the context of investigations and therapeutics of acne.

## 2 Anatomy/physiology of the sebaceous gland

**FIGURE 1:**
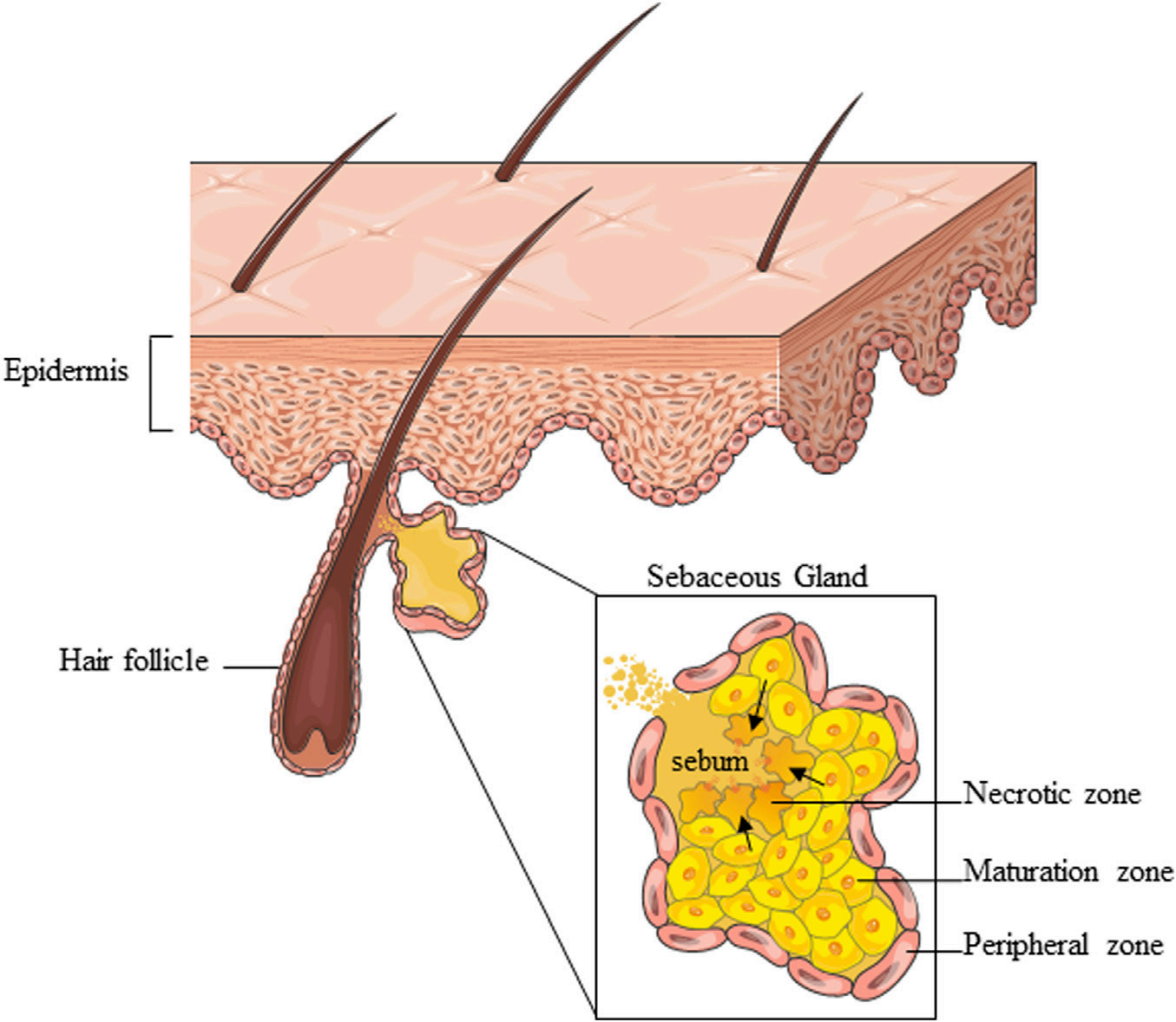
Schematic representation of the overall SG structure and partition into zones corresponding to various stages of sebocyte differentiation. Parts of Figure 1 were drawn using images from Servier Medical Art, provided by Servier, licensed under a Creative Commons Attribution 3.0 unported license.

The SG is a multi-acinar, multi-lobular holocrine gland bound to a hair shaft in the dermal layer of the skin except for the palms, soles, and dorsum of the feet (Zouboulis and Tsatsou, 2014). Each acinus empties into a duct, which converges with others to a common duct that tethers the hair follicle to form the pilo-sebaceous unit, which includes the hair, the hair follicle, and the erector pili muscle (Schneider et al., 2009; Zouboulis et al., 2016). The surrounding connective tissue sheath contains fibroblasts, small nerve fibers, and blood vessels, as well as components of the extracellular matrix (Clayton et al., 2019). There are also several examples of SGs “not associated with hair follicles” like those found at mucosal sites, including oral epithelium, perianal region, and eyelids (meibomian glands) (Ahmed et al., 2022). The SG comprises sebocytes around the lumen in a centripetally layered structure corresponding to various stages of differentiation of the sebocyte units (Figure 1). Undifferentiated and proliferative sebocytes populate the outermost part of the SG, called the peripheral zone. Differentiating sebocytes, which accumulate lipid droplets and dramatically increase cell size, reside in the maturation zone, adjacent to the peripheral one. Finally, in the necrotic zone, fully differentiated sebocytes disintegrate resulting in the holocrine secretion of sebum through the ducts. The sebocyte differentiation process takes around 7–14 days (Clayton et al., 2019; Clayton et al., 2020). Sebum is a complex mixture of different types of lipids, namely, triglycerides, fatty acids, wax esters, squalene, cholesterol esters, and cholesterol. Triglycerides and free fatty acids, taken together, account for the predominant proportion (40%–60%), followed by wax esters (20%–30%), squalene (10%–20%), and cholesterol and its esters (2%–10%). Squalene and wax esters are the most characteristic products of sebaceous secretion. Sebum is enriched in squalene, sebum-specific fatty acids, i.e., terminally branched-ones and the monounsaturated sapienic acid, and wax esters. Their exceptional amounts are unique to sebum and not found anywhere else in the body nor among the epidermal surface lipids (Pappas et al., 2009; Picardo et al., 2009; Camera et al., 2016). Sebum is a skin surface protectant serving several key functions in the skin, including lubrication, moisturization, and thermoregulation. By providing an antimicrobial and antioxidant barrier, sebum contributes to shielding environmental and pro-aging insults (Zouboulis et al., 2016; Clayton et al., 2020). Sebum synthesis is regulated by various mediators, which target the numerous regulatory receptors expressed by sebocytes and are consequently involved in the control of sebum secretion. The major receptors include the corticotrophin–releasing hormone (CRH) receptor, melanocortin receptors (MC-R1-5), opiate-R, androgen receptor (AR), growth hormone receptor (GHR), IGF-1 receptor (IGF-1R), insulin receptor (IR), retinoid X receptor (RXR), retinoic acid receptor (RAR), peroxisome proliferator-activated receptors (PPAR), neuropeptide Y receptor (NYR), etc., (Zouboulis et al., 2016). The multiplicity of interactions established between endogenous factors and the sebocytes ensures the continuous and the inherent activity of the SG under physiological conditions. Beyond the production of sebum, the SG presents intrinsic metabolic, neuroendocrine, and immunological functions, which places the SG among the key players in the multicellular network underlying skin homeostasis (Picardo et al., 2015; Zouboulis et al., 2016; Szollosi et al., 2018; Clayton et al., 2020).

## 3 Insulin production and mechanisms of action

Insulin synthesis and secretion is exclusive to the islet cells of the pancreas. The primary trigger of insulin secretion from the pancreas is glucose. Other factors like ketones, fatty acids, incretins like glucagon-like peptide (peptides released from the gut in response to food) can stimulate insulin release. After meals, insulin secretion increases once the concentration of glucose in the blood rises beyond 3.9 mmol/L. The insulin facilitates the upload of glucose from the blood into the tissue for storage in various forms like glycogen in the liver and muscles and lipids in the adipose tissue. Analogously to liver and adipose tissue, the SG accumulates glycogen. The glycogen-derived glycerophosphate has been addressed as a key player in the synthesis of sebum triacylglycerides by the SG from acetate (Downie and Kealey, 1998). Insulin ensures glucose entries cells and, at the same time, blood sugar remains within normal limit for maintaining body homeostasis (Raptis et al., 1975; Baumgard et al., 2016). Insulin virtually affects all the organs of the body by binding to the IR expressed at the cell surface. Insulin also binds IGF-1R but with less affinity (Giustina et al., 2015). The IR and IGF-1R are similar in their structure (Ullrich et al., 1986). In addition, there are insulin/IGF-1 hybrid receptors in most organs (Soos et al., 1990; Seely et al., 1995; Bailyes et al., 1997). These hybrid receptors have the capacity to dimerize and bind either insulin or IGF-1. The binding of insulin/IGF-1 to the receptors triggers the phosphatidylinositide-3-kinase (PI3K) second messenger pathway (Clemmons, 2012). This generates multiple effects in different tissues and organs. The overall effects are promoted cell growth, reduced blood glucose levels, increased glucose storage, protein synthesis and lipogenesis (Powers, 2012). In addition, insulin increases the serum levels of free IGF-1 by stimulating hepatic IGF-1 synthesis and suppressing the hepatic synthesis of IGFBP-1 (Clemmons, 2012).

## 4 Effect of insulin on the sebaceous gland

**FIGURE 2:**
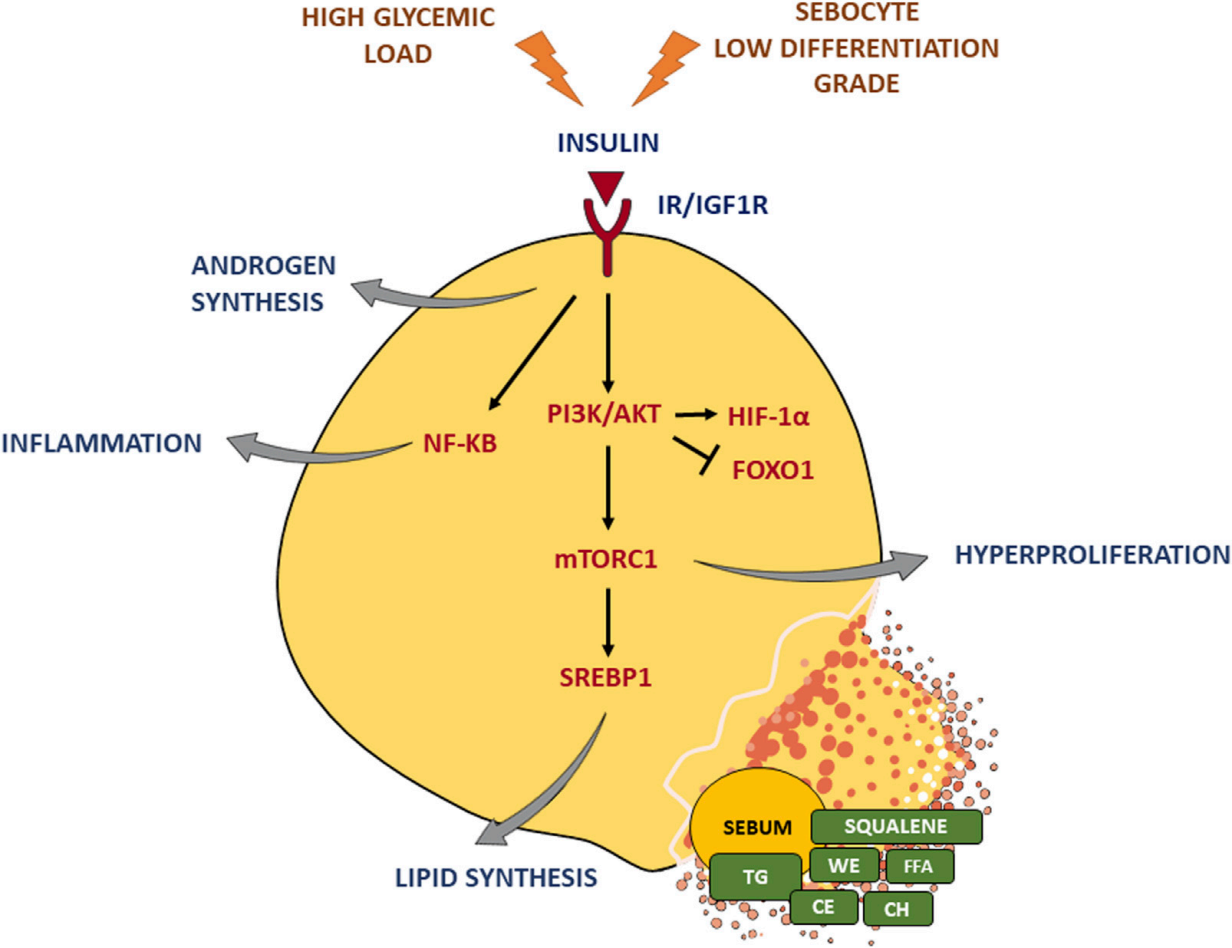
Insulin effects on the sebaceous gland. Insulin binding to IR/IGF-1R triggers the PI3K/Akt pathway and the critical downstream element mTORC1, which in turn activates the key lipogenic transcription factor SREBP1 and induces hyperproliferation. Moreover, insulin signaling enhances androgen synthesis as well as inflammatory response. A high glycemic diet and a sebocyte low differentiation grade exacerbate insulin stimulus. TG, triglycerides; FFA, free fatty acids; WE, wax esters; CE, cholesterol esters; CH, cholesterol. Parts of Figure 2 were drawn using images from Servier Medical Art, provided by Servier, licensed under a Creative Commons Attribution 3.0 unported license.

Insulin affects the function of the SG by inducing the increase in the size and number of sebocytes, as well as lipogenesis (Ding et al., 2015), through the binding to IR and IGF-1R and the consequent activation of the PI3K/Akt pathway (Mirdamadi et al., 2015; Briganti et al., 2020). The stimulation of IGF-1R is also enhanced by the incretion of free IGF-1 that occurs when insulin secretion increases. A critical downstream element in the Akt pathway is the mammalian target of rapamycin (mTOR) (Melnik and Schmitz, 2009; Huang and Fingar, 2014; Monfrecola et al., 2016), a nutrient-sensitive regulatory factor involved, through the mTOR complex 1 (mTORC1) (Wang and Proud, 2009), in the upregulation of the sterol response element binding protein-1 (SREBP-1) (Porstmann et al., 2009; Quinn and Baum, 2012) and the gamma isoform of PPAR (PPARγ) (Porstmann et al., 2009; Laplante and Sabatini, 2013), which are both involved in the lipid synthesis occurring in sebocytes (Ricoult and Manning, 2013). The activation of the PI3K/Akt pathway produced the concomitant reduction of the nuclear level of forkhead box class-O1 (FoxO1) transcription factor (Van Der Heide et al., 2004; Huang and Fingar, 2014), thus removing the suppression of SREBP1 (Smith et al., 2006; Smith et al., 2008b), PPARg (Trivedi et al., 2006; Dozsa et al., 2014), androgen receptors (AR) (Li and Al-Azzawi, 2009; Lai et al., 2012), liver X receptor (LXR) (Russell et al., 2007; Hong et al., 2008), leading to the further promotion of sebocyte lipogenesis, cell growth, and proliferation (Armoni et al., 2006; Fan et al., 2007; Fan et al., 2009). Furthermore, the PI3K/Akt signalling is able to activate hypoxia-inducible factor 1a (HIF-1a), a transcription factor involved also in lipid accumulation in sebocytes and the upregulation of the inflammatory mediators (Semenza, 2013; Choi et al., 2021) (Figure 2).

The effect of insulin on sebocytes is affected by the level of sebocyte differentiation. Low differentiated SZ95 sebocytes tend to have a higher basal expression of IGF-1R and IR at the protein level, associated with an upregulation of phospho-Akt (pAkT) and phospho-S6 (pS6) and a down-modulation of phosphatase and tensin homolog (PTEN) expression, compared to differentiated ones (Ottaviani et al., 2020). This leads to increased susceptibility to insulin stimulus of poorly differentiated SZ95 sebocytes.

## 5 Implications of insulin effect on the sebaceous gland

Diseases of the SG like acne vulgaris are related to abnormal hormonal stimulation of the gland. The reports of insulin resistance in patients with severe acne (Del Prete et al., 2012; Emiroglu et al., 2015; Nagpal et al., 2016) and the association of acne with diets delivering high glycaemic loads (Adebamowo et al., 2008; Smith et al., 2008a; Melnik, 2015) create a strong basis to examine the role of insulin in SG function. No acne has been found in non-Westernized populations still living under Paleolithic dietary conditions constraining carbohydrates, milk, and dairy products (Cordain et al., 2002; Melnik, 2018). On the other hand, consumption of dairy milk products has been associated with a higher frequency and severity of acne, as its components enhance the effects of insulin and IGF-1 on the production of androgen hormones and sebum and stimulate the formation of comedones (Bhate and Williams, 2014; Bronsnick et al., 2014; Albalwa et al., 2023). Acne manifests mostly at puberty, coincidentally with peaking androgens levels and insulin/IGF-1 signalling. Insulin reinforces the entire androgen axis: The pituitary gland, where it acts as a gonadotrophin amplifier; the gonads where it stimulates androgen synthesis; the adrenal glands, where it stimulates the production of androgenic precursors; the liver, where it inhibits the production of sex hormone binding globulin (SHBG), an important carrier for regulating androgen activity, and the skin, where SG is the major site of androgen biosynthesis (Chen et al., 2011; Nikolakis et al., 2016; Ju et al., 2017; Zhao et al., 2023). Insulin/IGF-1 and androgens trigger the mTOR pathway, which represents a central node between puberty, diet, and acne onset. In post-adolescence acne patients, increased glycaemia levels have been observed (Imperato-McGinley et al., 1993; Dozsa et al., 2014; Balta et al., 2015). The plasma glucose levels, which are directly associated with glucose intake, provide a carbon source for fatty acid synthesis (Strable and Ntambi, 2010; Lodhi et al., 2011) and stimulate the release of insulin and its signalling (Ameer et al., 2014), affecting *de novo* lipogenesis (DNL). DNL is a complex and highly regulated pathway that synthetizes fatty acids from exceeding carbohydrates (Ameer et al., 2014) and local DNL within SG is primarily responsible for human sebum production (Esler et al., 2019). Deregulated DNL is commonly associated with insulin resistance (Jensen-Urstad and Semenkovich, 2012). A low-glycaemic diet results in an improvement of insulin sensitivity and acne severity (Raptis et al., 1975; Imperato-McGinley et al., 1993; Ding et al., 2015) associated also with an improvement in the fatty acid composition of sebum (Smith et al., 2008b). The insulin/IGF1-dependent hyperstimulation of DNL promotes an increased release of fatty acids, leading to lipotoxicity, inflammation, and further promotion of the IR downstream signalling (Jensen-Urstad and Semenkovich, 2012; Ameer et al., 2014; Kim et al., 2017; Esler et al., 2019). Moreover, inflammatory cues contribute to creating a microenvironment facilitating the initiation and the aggravation of acne manifestations. The interplay of inflammatory cytokines and oxidated lipids, which are part of compositional modifications of acne sebum, concur to the acne lesions onset and progression (Capitanio et al., 2009; Capitanio et al., 2010; Ottaviani et al., 2010). This process also involves keratinocytes of the pilosebaceous duct, contributing to comedone formation due to increased proliferation of the infundibular epithelium (Zouboulis et al., 2014; Mastrofrancesco et al., 2017; Manfredini et al., 2019; Briganti et al., 2020). Acne may also be a common component of many systemic diseases or syndromes, which are also linked to insulin resistance (Chen et al., 2011). Patients with the polycystic ovarian syndrome (PCOS), hyperandrogenemia, insulin resistance, and acanthosis nigricans (HAIR-AN) syndrome, where hyperinsulinemia is present, also develop hyperseborrhea and acne (Elmer and George, 2001; Lowenstein, 2006; Kelekci et al., 2010). Acne may serve as a cutaneous marker for these syndromes. In metabolic diseases like type 2 diabetes mellitus and metabolic syndrome, characterized by insulin resistance (hyperinsulinemia) and increased mTORC1 signaling, acne may be a cutaneous feature (Nagpal et al., 2016; Melnik, 2018). There is a need for an improved dietary management of acne like in the other metabolic disorders, like type 2 diabetes mellitus, where balanced diets are essential. More studies are required to invigorate the connection between acne and these metabolic diseases. It is possible that in the future, acne will be viewed as a component of metabolic syndrome in adults.

## 6 Possible therapeutic strategies in acne

Due to the implication of a high glycemic diet and insulin/IGF-1 in the pathogenesis of acne, mechanisms of abatement of insulin level or action are useful targets in the treatment of this disorder. Sensitizers of the IR like the biguanides, have been evaluated as adjunct therapy in moderate to severe acne vulgaris. Metformin improves insulin sensitivity resulting in the increased uptake of glucose in peripheral tissues and inhibits the mTORC1 complex action (Fabbrocini et al., 2016; Lee and Smith, 2017; Albalat et al., 2022). Acetyl-Coenzyme A Carboxylase (ACC), which catalyzes the conversion of acetyl-CoA to malonyl-CoA, is the rate-limiting enzyme in DNL, a process highly responsive to insulin. The pharmacological inhibition of ACC, leading to the suppression of sebum production, may also be exploited as a potential therapeutic option for the treatment of acne (Esler et al., 2019). Another possible therapeutic strategy may target sebocytes with a low grade of differentiation, which present upregulated expression of IR and enhanced mTOR signalling, resulting in a higher susceptibility to insulin stimulus. Scientific evidence suggests a role of altered sebocyte differentiation in the development of acne (Ottaviani et al., 2020), in agreement with genetic data on the involvement of the differentiation process of the pilosebaceous unit in the pathogenic mechanisms (Barrault et al., 2015; Clayton et al., 2019; Common et al., 2019). Induction of differentiation in sebocytes by means of a PPARγ modulator has proven effective in abating the effects of the insulin challenge in sebocyte inflammation and sebogenesis. Topical application of a PPARγ modulator in acne patients has resulted in amelioration of clinical signs and improvement of the lipid balance in sebum (Mastrofrancesco et al., 2017; Ottaviani et al., 2020).

## 7 Conclusion

SG is an active partaker in the cell-cell cross talk in the skin. The SG activity is relevant to both physiological homeostasis and pathological processes. A plethora of autocrine and paracrine factors regulates the SG functions. Affecting the metabolic processes in the SG, insulin is a major hormonal trigger in acne development. Insulin induces an increase in the size and number of sebocytes, as well as lipogenesis and inflammatory response, contributing to the initiation and aggravation of acne manifestations. These effects, predominantly in low differentiated sebocytes, are essentially mediated through the Insulin/IGF-1 receptor and the PI3K/Akt signalling pathway. By increasing glucose loads and stimulating insulin secretion, high glycaemic diets worsen the effects of insulin/IGF-1 on the SG. In addition, acne is a common cutaneous feature of diseases and syndromes linked to insulin resistance and related hyperinsulinemia, such as PCOS. Acne could be viewed as a metabolic disease, or a component of the metabolic syndrome where dietary management is very important. A better understanding of the mechanisms of insulin action on the SG may stimulate the development of novel therapeutic strategies targeting specific molecular pathways in the management of acne.
